# A Behavioral Activation Mobile Health App for Smokers With Depression: Development and Pilot Evaluation in a Single-Arm Trial

**DOI:** 10.2196/13728

**Published:** 2019-11-27

**Authors:** Jaimee L Heffner, Noreen L Watson, Edit Serfozo, Kristin E Mull, Laura MacPherson, Melissa Gasser, Jonathan B Bricker

**Affiliations:** 1 Public Health Sciences Division Fred Hutchinson Cancer Research Center Seattle, WA United States; 2 Greenebaum Comprehensive Cancer Center Baltimore, MD United States; 3 University of Washington Seattle, WA United States

**Keywords:** tobacco, nicotine, smoking cessation, depression, smartphone

## Abstract

**Background:**

The integration of Behavioral Activation Treatment for Depression (BAT-D) into smoking cessation interventions is a promising approach to address depression as a barrier to quitting. However, this approach has only been tested as a face-to-face intervention, which has low reach.

**Objective:**

The aims of the study were to develop a BAT-D mobile health app with high potential reach and determine its feasibility, acceptability, and preliminary effects on theory-based behavioral processes of behavioral activation, reduced depressive symptoms, and smoking cessation.

**Methods:**

Following a user-centered design process consisting of competitive analysis, focus groups, and prototype testing, we conducted a single-arm pilot trial of Actify!, a BAT-D app for depressed smokers. Participants used SmokefreeTXT along with Actify! to provide cessation content that had not yet been built into the app for this initial phase of pilot testing. Participants in the trial were current, daily smokers with mild to moderate depressive symptoms. We examined use outcomes for all enrolled participants and process and cessation outcomes at 6 weeks postenrollment for study completers (16/17, 94% retention).

**Results:**

Regarding acceptability, average number of log-ins per participant was 16.6 (SD 13.7), and 63% (10/16) reported being satisfied overall with the app. Posttreatment interviews identified some usability challenges (eg, high perceived burden of planning and scheduling values-based activities). There was a significant decrease in depressive symptoms from baseline to follow-up (mean change in Patient Health Questionnaire–9 scores was –4.5, 95% CI –7.7 to –1.3; *P*=.01). Additionally, carbon monoxide (CO)-confirmed, 7-day point prevalence abstinence (PPA) at 6-week follow-up was 31% (5/16), and the 30-day PPA was 19% (3/16).

**Conclusions:**

Results demonstrate promising engagement with Actify! and potential for impact on theory-based change processes and cessation outcomes. Preliminary quit rates compare favorably to previous trials of smoking cessation apps for the general population (ie, short-term, self-reported 30-day quit rates in the 8% to 18% range) and a previous trial of face-to-face BAT-D for depressed smokers (ie, CO-confirmed, 7-day PPA rate of 17% at end of treatment).

## Introduction

Cigarette smoking remains the leading preventable cause of death in the United States as well as the leading cause of cancer-related mortality, accounting for 32% of all cancer deaths [1,2]. Only 6% of smokers each year are able to successfully quit [3]. Novel interventions are needed to target the key factors that maintain smoking, including depressive symptoms, which are present in 40% to 55% of treatment-seeking smokers [4,5]. Depressive symptoms at the time of a quit attempt, including high negative affect (ie, aversive internal states such as anxiety or sadness) and low positive affect (ie, reduced experience of pleasure or enthusiasm), reduce the odds of smoking cessation by as much as 50% [4,6-8]. Failure to address depressive symptomatology as a barrier to quitting is a significant problem with the current standard treatment approach.

Behavioral Activation Treatment for Depression (BAT-D) is a promising approach for improving smoking cessation outcomes among smokers with depressive symptoms [9]. The primary goal of BAT-D is to increase rewarding and valued experiences in daily activities by planning and scheduling these activities to increase their frequency. These changes in daily activities are expected to increase positive affect and reduce negative affect and avoidance behaviors [10]. BAT-D provides an integrative, behavioral theory–based approach to address depressive symptoms in the context of a smoking cessation intervention, as reinforcement theory holds that both smoking and depressed behaviors are maintained by limited reward for alternative, healthy behaviors [11]. In support of the theory, previous work has demonstrated that increased involvement in rewarding, nonsmoking behaviors (eg, physical activity, playing games with family) is associated with improved depressive symptoms and successful quitting [12].

Adding BAT-D to standard cessation counseling (eg, Behavioral Activation Treatment for Smoking, or BATS) can improve quit rates. In a pilot randomized controlled trial (RCT), smokers with depressive symptoms were assigned to receive either group-delivered BATS (n=35) or standard cessation treatment (n=33), and both arms received transdermal nicotine patches [9]. Participants were assessed at baseline, throughout treatment, and at 4, 16, and 26-week follow-ups for depressive symptoms and carbon monoxide (CO)–verified 7-day PPA. At 4 weeks postquit date (ie, end of behavioral treatment), BATS participants were 2.1 times more likely to be abstinent (*P*=.02) than standard treatment participants (17.1% vs 9.1%). As expected, abstinence rates declined over time in both groups, yet there remained a significant effect of BATS treatment at the 26-week follow-up (14.3% vs 0%; *P*=.02). Participants receiving BATS also evidenced greater improvements in depressive symptoms across the 26-week follow-up period. Another recently published pilot study showed promising results of a BATS-based cessation treatment targeted at smokers recently hospitalized with acute coronary syndrome (Behavioral Activation Treatment for Cardiac Smokers [BAT-CS]), a population with a high prevalence of depression [13]. Participants received either the BAT-CS intervention (n=28) or an intervention using standard cessation counseling (standard of care, n=31). Results of this feasibility trial indicated that BAT-CS was highly acceptable to participants and also produced greater improvements in positive affect, negative affect, and stress than standard-of-care counseling at 24-week follow-up. Additionally, the effect size for BAT-CS on smoking cessation was potentially promising: odds ratio 1.27 (95% CI 0.41 to 3.93) for 7-day PPA at 24 weeks. The study was not powered to detect statistically significant effects, but cessation and mood outcomes were all in the hypothesized direction, favoring BAT-CS over standard of care.

At present, BAT-D–based smoking cessation interventions have solely been tested as a face-to-face treatment, which would reach only 4% to 6% of smokers in the United States based on current use of the traditional treatment modalities of individual and group counseling [3,14,15]. We developed a BAT-D mobile health (mHealth) app for smoking cessation to extend the reach of this promising intervention to the estimated 13 to 16 million smokers with smartphones who have depressive symptoms [8,15-17], the vast majority of whom will not seek face-to-face counseling. BAT-D is a promising treatment approach for a targeted mHealth smoking cessation intervention because it has shown promise helping smokers with depressive symptoms quit smoking in the face-to-face format [9,13], its components are well suited to translation into an app (eg, scheduling valued activities and tracking their completion), and it has a strong foundation in behavioral theory [11]. Indeed, other groups have begun developing and testing BAT-D apps for depression either to be used in conjunction with therapist support [18,19] or in self-guided format and designed for a primary care context [20,21]. With the most advanced evaluation of a BAT-D app to date being pilot RCTs not adequately powered to test efficacy [18,22,23] and the remainder of the published work limited to intervention descriptions or single-arm feasibility studies [19-21], there remains much to be learned about how to effectively translate BAT-D principles into mHealth interventions.

In this report, we describe the development process and preliminary evaluation of Actify!—a BAT-D smoking cessation mHealth app for depressed smokers. The aims of the study were to develop an mHealth app with great potential for high reach built upon BAT-D principles and determine its feasibility, acceptability, and preliminary effects on theory-based behavioral processes of behavioral activation, depressive symptomatology reduction, and the primary outcome of smoking cessation.

## Methods

### Development Overview

With the support of a pilot grant from the Fred Hutchinson Cancer Research Center (principal investigator JLH), we developed a working prototype of a smartphone app, Actify!, that delivers BAT-D as part of a smoking cessation intervention. The treatment frameworks guiding the app development were the BAT-D manual by Lejuez et al [10] for the treatment of depression [10] and the BATS integrative treatment for smoking cessation and depression by McPherson et al [9]. Key components of these treatments that were included in the app were (1) providing the treatment rationale for behavioral activation, (2) identifying values (ie, developing awareness of what matters most to each individual), (3) planning and scheduling values-based activities that are important or enjoyable as a means of increasing the frequency of these activities, and (4) monitoring progress at implementing values-based activities. Although they are included in the treatment manuals for face-to-face BAT-D, we decided that the initial version of Actify! would not include detailed, hour-by-hour monitoring of daily activities (including weeks where intervention activity is limited to self-monitoring) and formal social contracts for completing activities, as we learned from early user research that minimizing data entry burden (for daily monitoring) and privacy concerns (for social contracts) were critical design requirements. However, we did create content that prompts users, in a less structured manner, to consider how important or enjoyable their current daily activities are and reach out for social support as a means of overcoming barriers to completing activities.

The app was created following an iterative user-centered design process [24] including competitive analysis, focus groups, and usability testing of low- and high-fidelity prototypes followed by a single-arm pilot trial.

### User-Centered Design Process

We first conducted a competitive analysis by reviewing a small number (5-6) of both paid and free apps and Web-based programs (eg, Mood Coach, Joyable) for depression as well as apps that include elements similar to the components of behavioral activation, such as scheduling and tracking activities or apps for setting goals and marking them complete (eg, Iwish, Habitica). The purpose of a competitive analysis is to review other programs currently on the market to generate (via the personal experience of the design team) and assess (via user comments in app stores) potential user sentiment toward them in relation to their design and functionality. Such information is useful as part of the ideation phase of development to understand desirable features for a new app as well as pain points (ie, problems that reduce the quality of the user’s experience). Examples of desirable features identified through the competitive analysis to reduce pain points were making valued activities customizable and offering menus of options to keep text entry fields to a minimum and reduce user burden.

Two focus groups of 4 and 6 persons were conducted in November 2016 to assess potential users’ previous experiences using depression apps (eg, likes and dislikes); develop user personas, fictional characters based on typical users, a common user-centered design strategy [25]; elicit ideas for valued activities in several life domains (eg, relationships, health, leisure) the app could suggest to the user; and understand users’ associations between emotions and possible color palettes that might appear in the app. User personas were developed in order to guide design decisions (Multimedia Appendix 1), and lists of common values (eg, having close relationships, being a good citizen/community member) and valued activities (eg, helping others, visiting friends and family) were generated for inclusion in the app. Focus group data were then used to generate two distinct prototypes of a BAT-D app. We used prototyping software to allow user research participants to interact with these prototypes on a smartphone in order to provide feedback on design and functionality. Individual interviews were conducted to evaluate each prototype (7 for prototype A and 6 for prototype B).

Based on users’ reactions to these low-fidelity prototypes and general satisfaction with the interface concept of prototype B (vs more mixed feedback about prototype A), the study team decided to continue developing that prototype for the first version of Actify! The user experience design team then conducted three iterative rounds of testing between January and March 2017 with higher fidelity prototypes. In each round, users were given 4 to 5 tasks to complete. Through these tasks, the study team tested users’ ability to navigate the major app components of onboarding, values identification, activity scheduling, activity tracking, calendar, progress, and resources. These rounds of testing also included assessments of users’ expectations and desires for app functionality. Refinements to the app at this phase focused on more fine-grained details such as text size and readability, size of visual elements (eg, buttons), and clarity of instructions included in the app onboarding screens. Development of the programmed version of the app was completed by the study team’s programming partner, 2Morrow, in July 2017. For evaluation purposes, Actify! was developed as a native app for Apple’s iOS operating system. We chose a native app (as opposed to a Web app) for two main reasons: the option of potentially including features restricted to native apps (eg, interactive push notifications) and better responsiveness of native apps compared with Web apps (ie, less wait time between user action and app response). Supporting our choice to develop for iOS, a recent study demonstrated that the majority (77%) of individuals who download smoking cessation apps download them on iOS devices as opposed to Android (23%) [26]. However, we plan to develop later versions of Actify! so that it is accessible on multiple platforms.

### Description of Actify!

**Figure 1:**
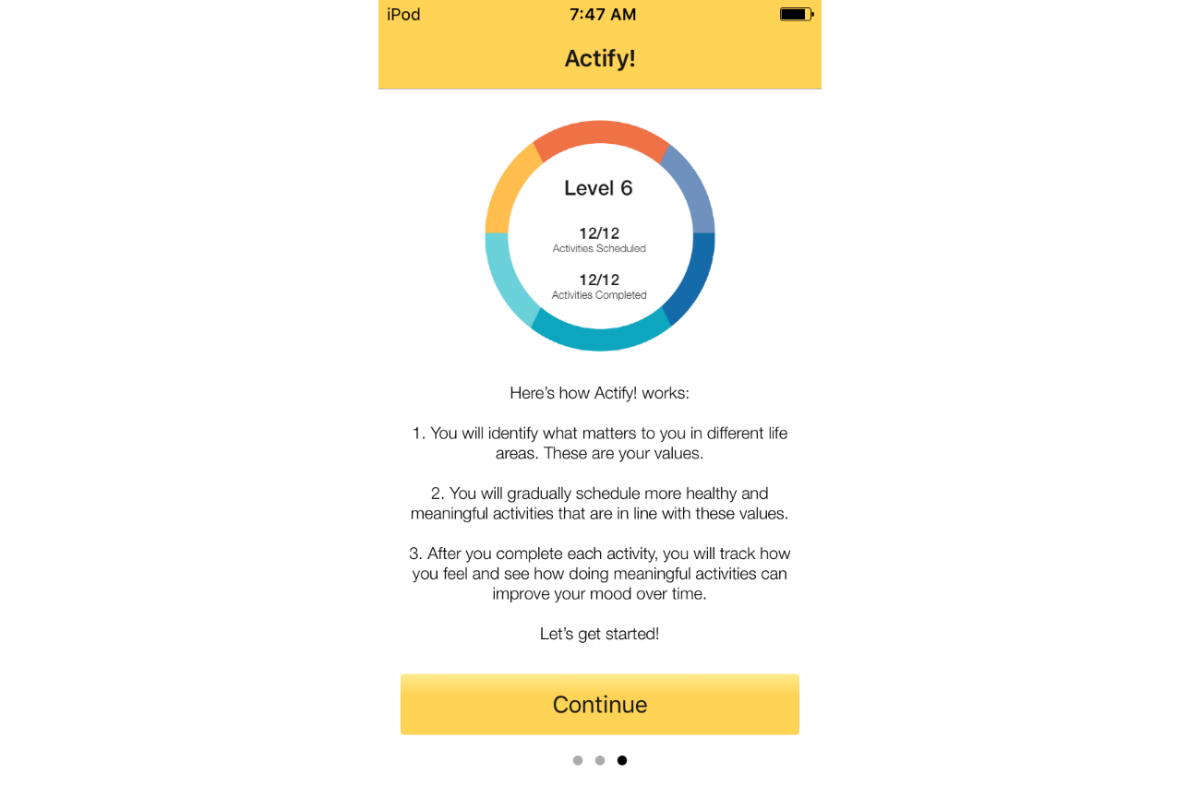
Onboarding.

**Figure 2:**
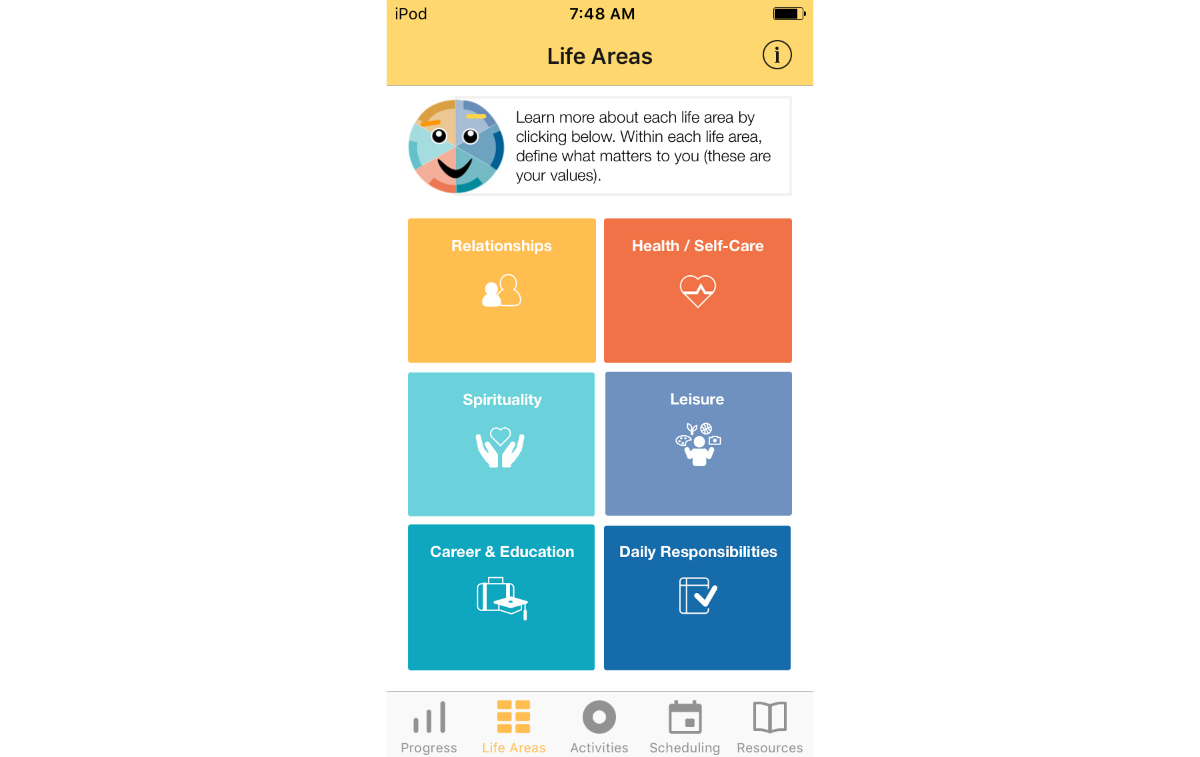
Life areas.

**Figure 3:**
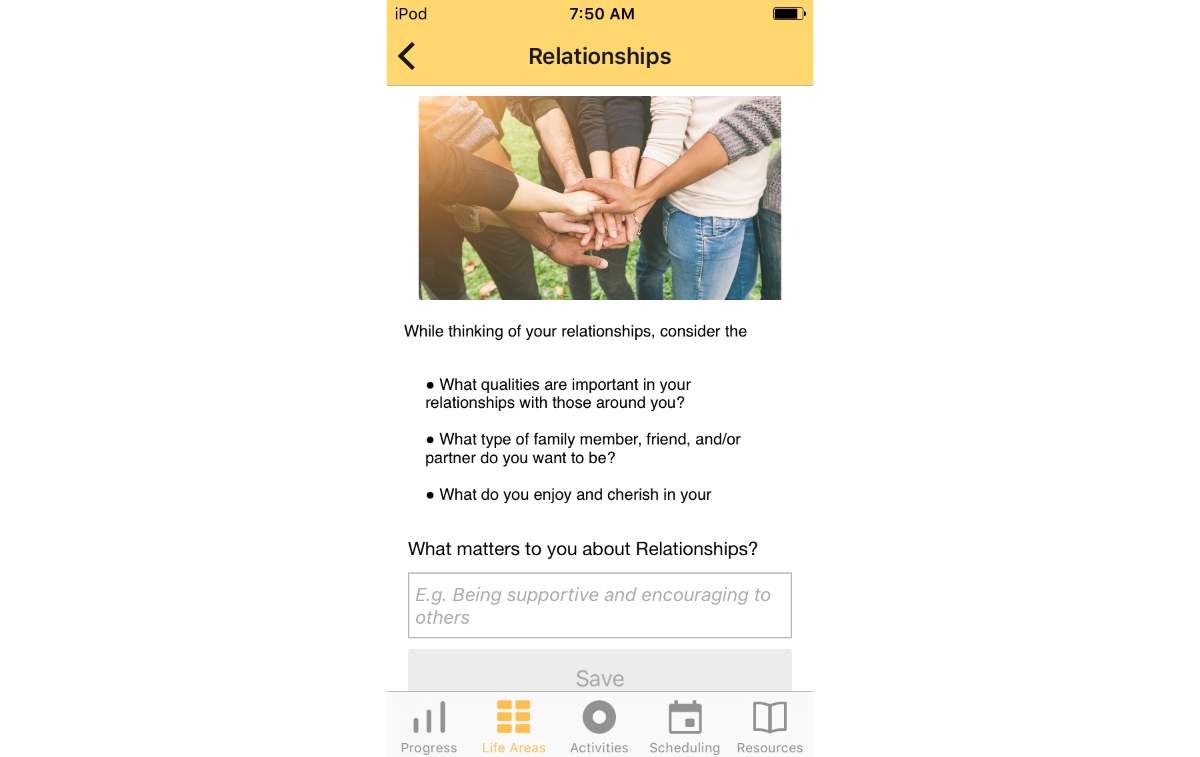
Values identification within life areas.

**Figure 4:**
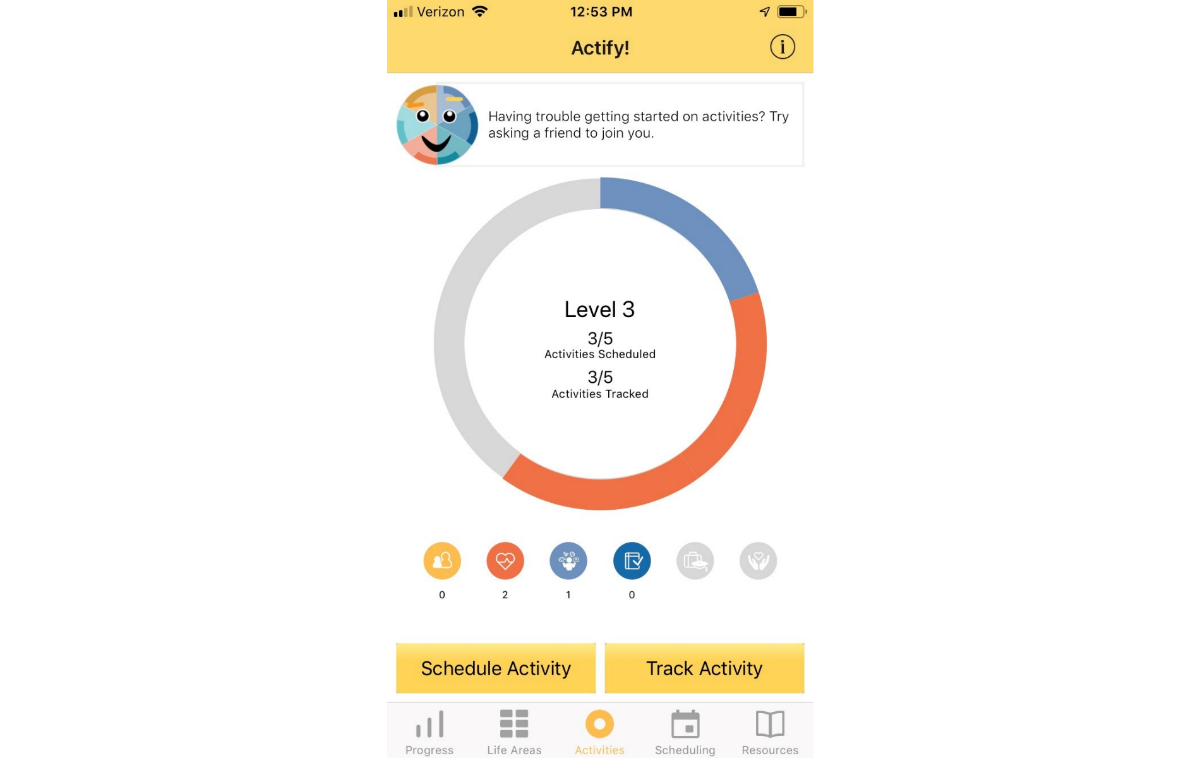
Activity wheel.

**Figure 5:**
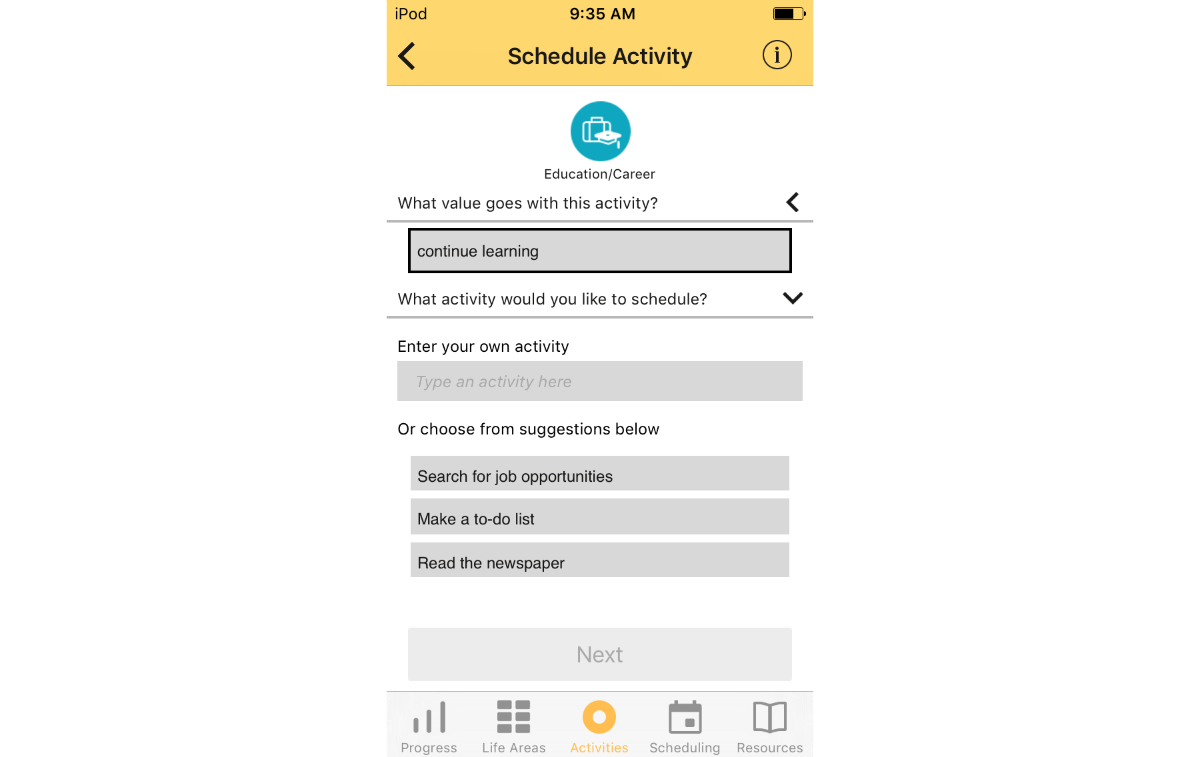
Scheduling.

**Figure 6:**
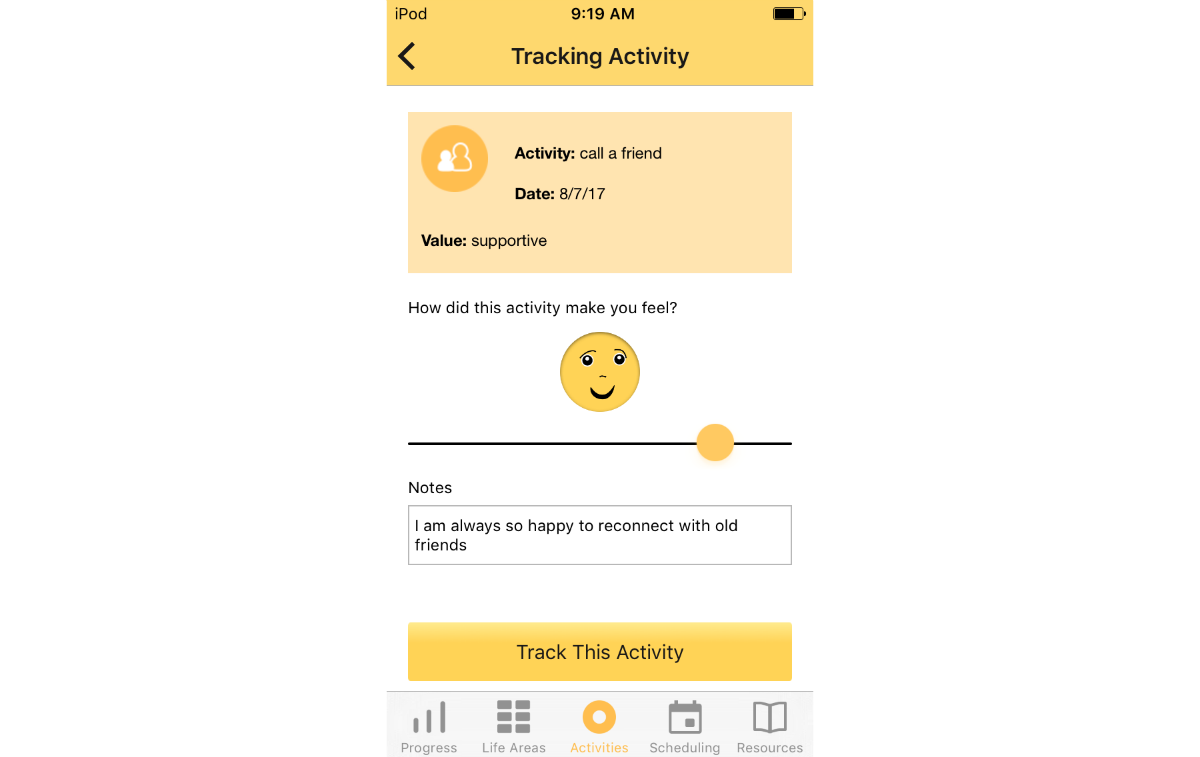
Tracking.

**Figure 7:**
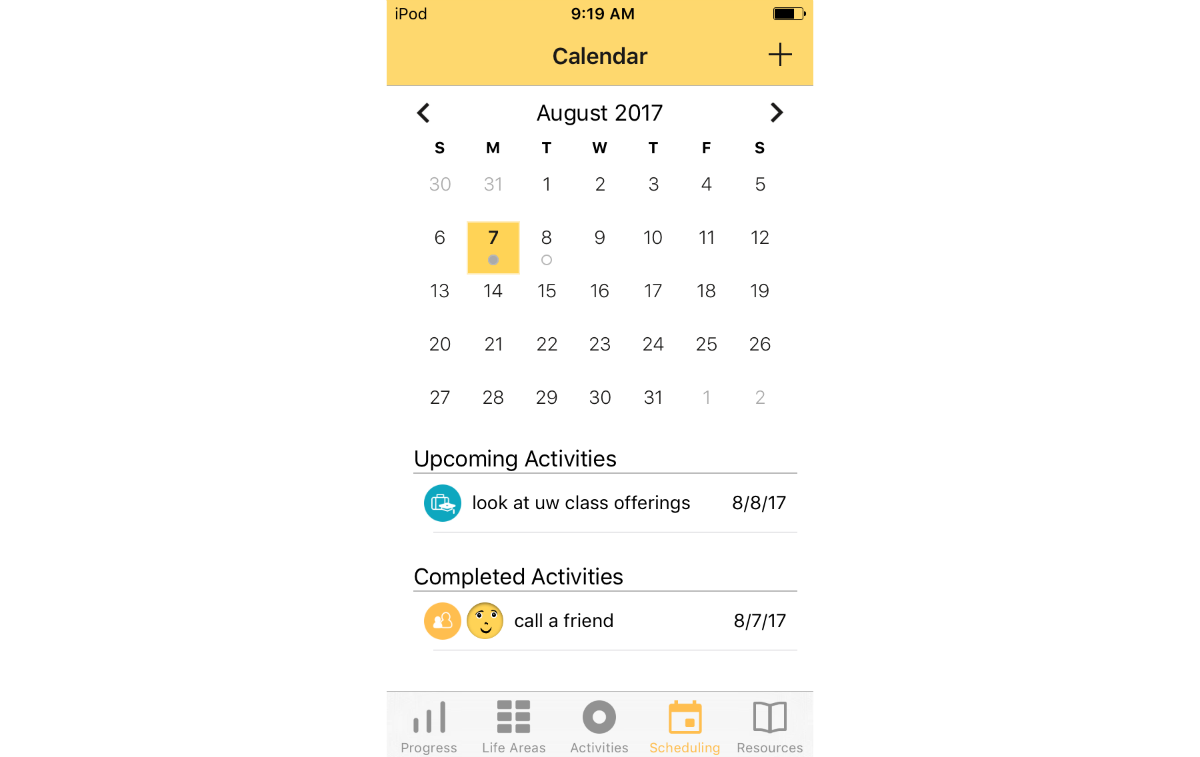
Calendar.

**Figure 8:**
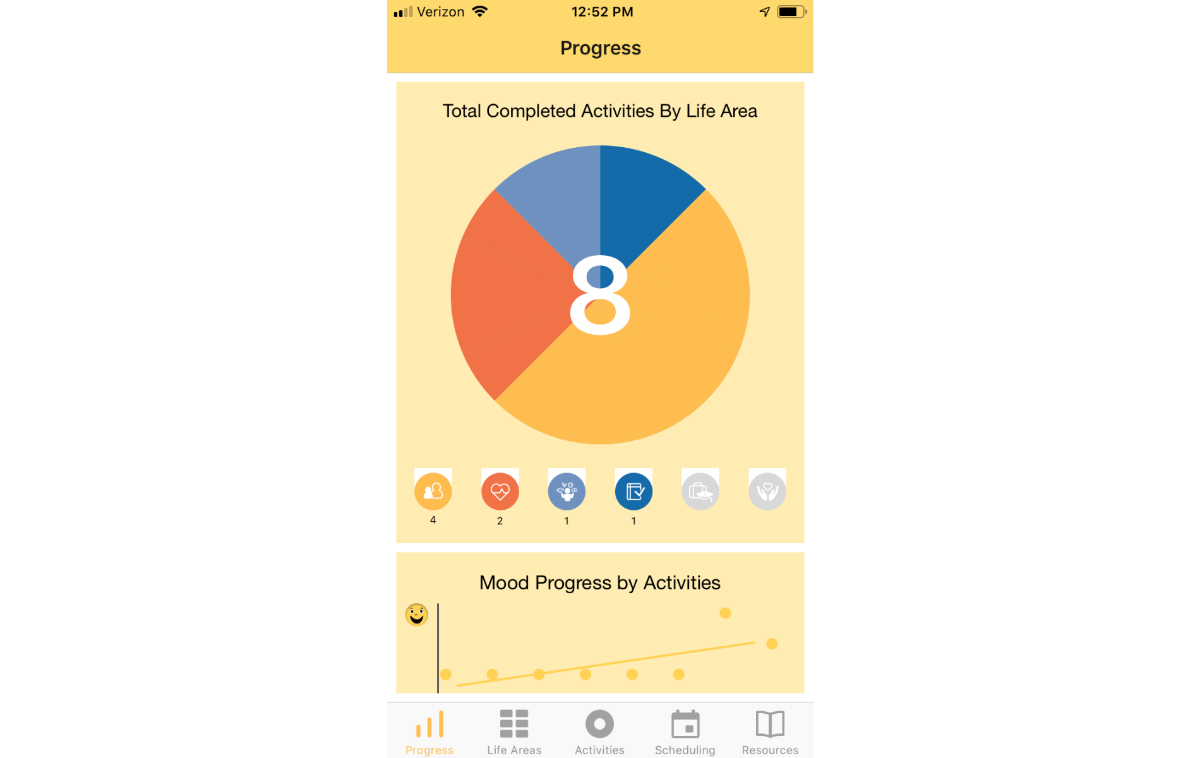
Progress.

Guided by the BAT-D treatment model of Lejuez and colleagues [10] as well as the BATS smoking cessation protocol developed by author LM and found to show promise in a pilot RCT [9], the core functions of the current version of Actify! are as follows:

- Treatment rationale: introducing the user to the program by describing how behavioral activation works and providing instructions on how to use the program (Figure 1)
- Values identification: helping the user identify what matters most to them (eg, family, fun) within six core life areas—relationships, health/self-care, spirituality, leisure, career and education, and daily responsibilities (Figures 2 and 3)
- Activity scheduling: planning and scheduling of values-based activities that are important or enjoyable (eg, reading to my children) and organized by life area (Figures 4 and 5), including life area-specific activity suggestions (ie, a bank of 10 to 20 suggestions for each) and the ability to set an alert for each scheduled activity
- Progress monitoring: tracking completed activities and mood (Figure 6) and visualizing overall progress toward meeting activity goals in calendar form (Figure 7) and across time, broken down by life area (Figure 8)

The app also contains a Resource section with Frequently Asked Questions (eg, “What if I increase my activity and still don’t feel better?”) as well as a Depression Help section (eg, “What is depression?” and “What if I am thinking of harming myself?”).

To increase engagement, the app has a game-like structure [27] with a level system that, over time, encourages escalating activity goals and increasing breadth of life areas represented in the chosen activities. For the pilot trial, 6 levels were available. Level 1 encourages users to complete 2 activities in one life area. By Level 6, the user has been prompted to identify values in all 6 life areas and must complete a total of 12 activities to finish the level. Each level begins with a user story of a person who improved their mood and quit smoking using the program. User stories illustrating how values can be translated into actions were associated with cessation in our prior work [28]. We also used the content of user stories to address common barriers to implementing behavioral activation, such as setting goals that aren’t realistic, having difficulty reaching out to others for support, and feeling overwhelmed and having difficulty getting started. An example user story is that of Elisa: “A clean home makes me feel proud and like I have self-respect. But feeling depressed, I let my house get really messy. I felt overwhelmed by all of the cleaning I needed to do. I put washing dishes in my schedule the first night. I felt so much better afterwards and started scheduling more things to clean.” Actify! also includes motivational messages that appear each time the user opens the home screen (eg, “Having trouble getting started on activities? Try asking a friend to join you”) and notifications that appear in response to certain user action or inaction (eg, a suggestion to try new activities if the user enters three consecutive low mood ratings; a suggestion to start with small activities if three consecutive scheduled activities are not tracked as being completed; a notification to suggest scheduling an activity after three consecutive days of no activity scheduling or tracking).

Because the other components to support smoking cessation were not included in this early development phase due to budgetary constraints, participants used the SmokefreeTXT text messaging program from the US National Cancer Institute (NCI) alongside Actify! to provide content similar to what will be included in the later versions of the app. SmokefreeTXT was developed by NCI’s Smokefree.gov development team and includes 6 weeks of daily messages delivered 2 to 4 times per day, with more frequent messaging on and around the quit date. Messages are designed to prepare smokers for their quit day and support cessation maintenance. Some messages are interactive, prompting users to respond to a query (eg, “To deal with cravings: breathe in, hold for 5 seconds, breathe out, and repeat. What is your current craving level? Reply: HI, MED, LOW”). Programmed messages provide tailored feedback based on the user’s responses to these queries.

### Pilot Trial Procedures

#### Trial Overview

Between November 2017 and June 2018 we completed a single-arm pilot trial of the initial programmed version of Actify! with functionality limited to the BAT-D component. Primary aims were to obtain preliminary data on user engagement and satisfaction and evaluate effects on the target behaviors of increased engagement in valued activities, smoking cessation, and improved mood. To achieve these aims, we conducted a 6-week evaluation to assess engagement and short-term efficacy outcomes and obtain detailed user feedback about the intervention.

#### Recruitment and Informed Consent

Participants were recruited from the Seattle area primarily via no-cost Craigslist ads (53% of sample) and paid Facebook advertisements (41% of sample). Interested potential participants were screened for participation using a 2-stage process. First they completed a Web-based preliminary screening form that assessed only the nonsensitive eligibility criteria (ie, all selection criteria except questions about mental health). Those who were potentially eligible based on the initial screening were asked for contact information to follow up with a phone call from trained research staff. During this call, the purpose and procedures of the study were explained in further detail and remainder of the eligibility criteria (ie, the mental health–related criteria) were assessed. Those who remained interested and eligible were invited to participate in the study; those who were not eligible were given referral information for smoking cessation assistance. Eligible participants were invited to attend an initial face-to-face meeting, during which the full details of the study were explained and participants provided informed consent. Participants were instructed to download the Actify! app during the initial meeting and enroll in the SmokefreeTXT messaging program. They were encouraged to use the program as much as possible but told that the frequency and duration of use was ultimately their choice. The study was reviewed and approved by the Institutional Review Board of the Fred Hutchinson Cancer Research Center.

#### Participants

Inclusion criteria for the single-arm pilot trial were (1) age 18 or older, (2) current smoker, averaging at least 5 cigarettes per day for the last 30 days, (3) interested in quitting smoking in the next 30 days, (4) own an iPhone version 5 or higher (necessary to run the current version of Actify! without technical problems), (5) have experience downloading and using one or more apps on their iPhone, and (6) screen positive for mild to moderate current depressive symptoms (9-item Patient Health Questionnaire [PHQ-9] score 5-19 [29]). Exclusion criteria were (1) currently using a depression app or receiving other treatment for depression, including psychotherapy or medication as this would be a significant source of bias in a single-arm assessment of intervention effects, (2) mood symptoms too severe to be addressed with a self-guided app (ie, severe depression [PHQ-9>20] or current suicidal ideation [PHQ-9 item 9 score >1]), (3) receiving other treatment for smoking cessation, or (4) previous use of the SmokefreeTXT program.

#### Measures

Demographic items assessed at baseline included age, gender, education, employment, income, and marital status. The 6-item Fagerström Test for Nicotine Dependence (FTND) [30] assessed degree of physical dependence on nicotine. The Patient Health Questionnaire–9 (PHQ-9) is a widely used assessment of depressive symptoms that has demonstrated ability to detect symptom changes over time as a result of treatment [29]. The scale scores range from 1 to 27, with severity thresholds as follows: 5-9=mild, 10-14=moderate, 15-19=moderately severe, 20-27=severe. A score of 10 has 88% sensitivity and 88% specificity for a diagnosis of major depression [31]. The smoking timeline follow-back (TLFB) [32] is a calendar-based method of obtaining retrospective estimates of daily smoking. For establishing baseline smoking levels, the 30-day period prior to the baseline visit was used.

Treatment acceptability was assessed at the 6-week follow-up visit using measures of treatment use and satisfaction. The primary outcome of treatment use was defined as the number of times Actify! was opened on the phone, determined objectively via time-stamped, server-recorded page views. App page views separated by 10 minutes or more were counted as unique log-ins. Treatment satisfaction was assessed with 8 study-specific items on the 6-week outcome survey. Example items include “Overall, how satisfied are you with the Actify! app?” and “Overall, how useful was the app for helping you engage more in activities that are important to you?” Follow-up questions probed satisfaction with specific components of the program (eg, app organization, ease of use, and usefulness of text messages).

Efficacy for increasing behavioral activation was assessed using the 25-item Behavioral Activation for Depression Scale (BADS) [33], which is designed to detect changes in activation resulting from treatment. Scores for individual items range from 0 to 6, with total scores ranging from 0 to 150. Scores on the 7-item activation subscale range from 0 to 42. Efficacy for reducing depression symptoms was assessed via change in PHQ-9 scores. Scores for individual items range from 0 to 3, with total scores ranging from 0 to 27. Efficacy for smoking cessation was CO-confirmed 7-day PPA at 6 weeks postenrollment. The secondary efficacy end point was 30-day PPA at 6 weeks postenrollment. Smoking outcomes were assessed using the smoking TLFB [32]. Expired-air CO levels were taken at baseline and at the follow-up visit as a means of biochemically verifying smoking self-report. Consistent with the recommendations of the Society for Research on Nicotine and Tobacco Workgroup on Biochemical Verification [34], a CO cutoff of ≤8 ppm was used as a basis for confirming self-reported abstinence.

#### Adverse Events

Any adverse events volunteered by participants at the 6-week follow-up visit were recorded.

#### User Experience Interview

We collected qualitative data on participant experiences with the intervention via a semistructured interview (approximately 45 minutes) conducted at the 6-week follow-up visit. Objectives were to evaluate the (1) context of use, including time of day the app was used, what prompted the user to open the app, and how the user tended to interact with the app; (2) satisfaction with the user interface and app design, including the aesthetics, ease of use, and specific BAT-D components such as activity scheduling and tracking; and, (3) perceived effects of the app on behavior and mood. All interviews were completed by the first author, and detailed notes were recorded.

#### Follow-Up Procedures and Compensation

The 6-week follow-up was an in-person visit of approximately 1 hour that was scheduled at the time of the baseline visit. Participants received reminder calls and/or emails (based on individual preference) to increase adherence to their scheduled appointments. Compensation for completing the follow-up visit was higher than for the baseline visit ($70 vs $30) due to the greater length of the follow-up visit.

#### Statistical Analysis Plan

Consistent with the developmental nature of the study, statistical analyses were primarily descriptive. Changes in behavioral activation and depression symptoms from baseline to 6-week follow-up were evaluated using paired *t* tests, with *P*<.05 as the threshold for statistical significance.

## Results

### Recruitment, Retention, and Sample Description

We prescreened 250 individuals via Web-based survey to identify 177 potentially eligible participants. We were able to reach 74 of these individuals by phone to complete additional screening and enrolled 17 eligible participants in the single-arm trial. The most common reasons for ineligibility on the Web-based survey were not providing a name or contact information (73/250), not owning an iPhone 5 or higher (32/250), and smoking fewer than 5 cigarettes per day (20/250). On the phone screen, the most common reasons for ineligibility were inability to attend in-person visits (15/74, of which 14/74 were due to a Craigslist technical error that resulted in the ad being posted in multiple cities), currently receiving other treatment for depression (13/74), and not owning an iPhone 5 or higher (7/74).

Of the enrolled participants, 94% (16/17) completed the in-person 6-week follow-up visit and are included in the subsequent complete-case results. These participants were 50% (8/16) men, 38% (6/16) racial minority (two or more races [4/16], Asian [1/16], or other [1/16]), and 25% (4/16) Hispanic, with a mean age of 36.1 (SD 9.7) years. The majority identified as heterosexual (14/16, 88%), never married (10/16, 63%), employed (12/16, 75%), and had a bachelor’s degree or higher level of educational attainment (11/16, 69%). Average smoking at baseline was 6.5 (SD 3.8) cigarettes per day, and average FTND score was 2.6 (SD 2.1), indicative of low nicotine dependence. Severity of depressive symptoms at baseline was moderate, on average PHQ-9 10.2 (SD 5.0).

### Engagement and Satisfaction

As shown in Table 1, the average number of app openings per participant was 16.6 (SD 13.7) over the 6-week study period, with a minimum of 6, a maximum of 55, and a median of 13.

**Table 1:** Actify! use metrics for enrolled participants (n=17).

Characteristic		App use			Retention^a^, %	Rolling retention^b^, %
		Mean (SD)	Median	Range		
**Overall use**						
	Number of log-ins	16.6 (13.7)	13	6-55	—^c^	—
	Time per session (minutes)	4.2 (2.3)	4.5	0.7-8.5	—	—
	Unique number of days of use	10.9 (8.8)	8	4-37	—	—
	Log-ins, week 1	5.7 (3.2)	5	1-12	100	100
	Log-ins, week 2	2.4 (3.0)	1	0-9	71	94
	Log-ins, week 3	1.2 (1.3)	1	0-4	59	88
	Log-ins, week 4	1.6 (2.6)	0	0-9	47	65
	Log-ins, week 5	1.0 (2.4)	0	0-10	41	53
	Log-ins, week 6	0.8 (1.8)	0	0-7	35	35
**App component use**						
	Progress screen viewing	13.5 (14.2)	9	1-57	—	—
	Resources section viewing	2.5 (3.0)	1	0-8	—	—
	Calendar viewing	13.9 (14.3)	8	1-54	—	—
	Life areas added (maximum possible: 6)	4.8 (1.6)	5	0-6	—	—
	Activity scheduling	18.2 (23.9)	10	0-93	—	—
	Activity tracking	15.4 (23.3)	9	0-93	—	—

^a^Retention: proportion of users who logged in at least once that week.

^b^Rolling retention: proportion of users who logged at least once that week or in subsequent weeks.

^c^Not applicable.

The average numbers of times users accessed major components of the app are listed in Table 1 along with the medians and ranges for each metric. As shown in the table, all areas of the app were accessed at least once, on average, and the most-accessed features were activity scheduling (mean 18.2 [SD 23.9], median 10) and activity tracking (mean 15.4 [SD 23.3], median 9). Week-by-week retention (ie, proportion of users accessing the app at least once that week) and rolling retention (ie, proportion of users accessing the app at least once that week or in a subsequent week) are also provided in Table 1. As 30-day rolling retention is a commonly reported metric among app developers, the most comparable metrics for Actify! are a rolling retention of 65% (11/17) for week 4 and 53% (9/17) for week 5.

On the measure of treatment satisfaction, 63% (10/16) reported being satisfied overall, 63% (10/16) thought that the app was useful for increasing important activities, 63% (10/16) thought the app was organized, and 75% (12/16) found the accompanying text messages helpful.

### Efficacy for Smoking Cessation

As shown in Table 2, the CO-confirmed 7-day PPA rate at 6-week follow-up was 31% (5/16), and the 30-day PPA at 6-week follow-up was 19% (3/16). In a post hoc sensitivity analysis using a conservative cutoff for CO levels indicative of abstinence (3 ppm or less [35]), the findings were unchanged.

### Efficacy for Decreasing Depressive Symptoms and Increasing Behavioral Activation

Average change in depression on the PHQ-9 was a 5-point decrease from baseline (mean –4.5, 95% CI –7.7 to –1.3), which was a statistically significant improvement in symptoms (*P*=.01) as well as a clinically significant change from moderate symptoms to mild symptoms (Table 2). Average change in BADS overall scale scores and activation subscale scores (BAT-D’s theory-based change mechanism) were not statistically significant but were in the hypothesized direction: BADS total scores increased by 14 points (mean 14.4, 95% CI –2.6 to 31.3; *P*=.09) and activation subscale scores increased by 4 points (mean 3.6, 95% CI –1.7 to 8.9; *P*=.17; Table 2).

**Table 2:** Actify! cessation and process outcomes for the complete-case sample (n=16).

Variable		Value	*P* value
**Biochemically confirmed cessation at end of treatment**			
	7-day PPA^a^, complete case (n=16), n (%)	5 (31)	—^b^
	30-day PPA, complete case, n (%)	3 (19)	—
**Mechanisms of change**			
	Change in BADS^c^ activation subscale, mean (95% CI)	3.6 (–1.7 to 8.9)	.17
	Change in BADS total, mean (95% CI)	14.4 (–2.6 to 31.3)	.09
	Change in PHQ-9^d^, mean (95% CI)	–4.5 (–7.7 to –1.3)	.009

^a^PPA: point prevalence abstinence.

^b^Not applicable.

^c^BADS: Behavioral Activation for Depression Scale.

^d^PHQ-9: 9-item Patient Health Questionnaire.

### Adverse Events

No adverse events were reported during the 6-week evaluation period.

### User Experience Interview Findings

Regarding context of use, participants tended to interact with the app at home, either in the morning or the evening. They liked the concept of the app (ie, thinking about what’s most important to them and scheduling activities in line with those values) but indicated that implementation could be improved. The vast majority of user-identified pain points were in setting up the hierarchy of life areas, values, and activities to be scheduled. This hierarchical process, which was integrated into the scheduling of each activity (Figure 5), was perceived as overly burdensome and sometimes confusing to use. Participants had considerable difficulty distinguishing values from activities and therefore became confused when prompted to identify a value and select an activity that goes with that value. Some participants indicated that they scheduled and tracked activities at the time the activity was completed rather than prescheduling and tracking later, which was the intent of the intervention. They also wanted more activity suggestions (beyond the 3 they were offered each time an activity was scheduled) and the option to schedule repeat activities and/or select from a group of saved activities. Because of the data entry burden, some participants reported that they stopped using the app and switched to low-tech methods of scheduling and tracking activities (eg, writing activities on a calendar and using different highlighter colors to represent different life areas).

None of the participants could recall any specific detail of the introduction, which provided the treatment rationale and instructions for using the program, and many indicated that they didn’t remember seeing any introduction. Feedback on other specific app features was mixed. For example, some users liked the level system, noting that it helped create a sense of playfulness and reward as well as a feeling of accomplishment, whereas others didn’t find the level system engaging or motivating.

## Discussion

### Principal Findings

Collectively, our results demonstrate promising engagement with the app and a potential signal for impact on theory-based change processes, depression symptoms, and cessation outcomes. Taken together with the results of two published pilot studies on BAT-D for smoking cessation [9,13], these findings support the feasibility of a BAT-D app to improve smoking cessation outcomes and mood among smokers with mild to moderate depression. They also support future planned work to complete development of Actify!, modify it to improve usability, and evaluate the complete intervention in a controlled pilot trial.

No prior studies have investigated the efficacy of smoking cessation apps for depressed smokers; however, our efficacy data compare favorably to that of previous reports of smoking cessation apps more broadly as well as face-to-face BAT-D for cessation. Although a fully powered effectiveness trial has yet to be completed, mHealth apps for smoking cessation have been evaluated in a number of pilot studies, demonstrating encouraging results for acceptability and quit rates [36-42]. The literature base to support apps for smoking cessation is still emerging and currently includes only two RCTs—both of which are pilot trials with wide confidence intervals for the point estimates of cessation. In a pilot RCT comparing SmartQuit to the NCI’s QuitGuide app (n=196), 30-day quit rates at 2 months were 13% (95% CI 6% to 22%) in SmartQuit versus 8% (95% CI 3% to 16%) in QuitGuide [36]. In a pilot trial comparing a text message intervention to the REQ-Mobile smartphone app (n=102) for young adult smokers, the REQ-Mobile app produced a 30-day PPA rate of 18% (95% CI 7% to 28%) at 12 weeks compared with the text message quit rate of 31% (95% CI 18% to 45%) [38]. Compared with previous pilot trial data showing short-term, 30-day quit rates of 8% to 18% in a general population of smokers, our 30-day quit rate of 19% is encouraging, especially given that the Actify! quit rates represent biochemically confirmed abstinence, whereas the two published RCTs did not include biochemical verification of abstinence to validate self-reported cessation (nor did the majority of the observational and single-arm pilot feasibility studies). Although biochemical confirmation is not universally necessary or feasible for population-level intervention trials [34], an assessment of biochemically confirmed quit rates for cessation apps provides support for the validity of the findings. We also note that biochemically confirmed abstinence rates (ie, 19% for 30-day PPA and the more comparable 31% for 7-day PPA) for Actify! at 6 weeks postenrollment compare favorably with short-term cessation outcomes for the face-to-face study of Behavioral Activation Treatment for Smoking (BATS) for depressed smokers (ie, 17% biochemically confirmed 7-day PPA at 4 weeks postquit date) [9]. Finally, given that previous work has reported a 6-week end-of-treatment quit rate of 7% for SmokefreeTXT [43], the text messaging component alone seems unlikely to fully account for the quit rate of the Actify! program.

### Limitations and Future Directions

As an early pilot trial of an mHealth app, this study has a number of limitations. The sample size and study design (eg, single-arm pilot, n=16 in complete case analysis, no control group), albeit consistent with recommendations for early pilot intervention work [44], do not allow for strong causal conclusions about efficacy to be made, and estimates of cessation outcomes are inherently less reliable than would be the case in larger studies. We did not track nonstudy treatment use, so we cannot determine the extent to which use of other treatments may have influenced outcomes. We also used an existing text message intervention (SmokefreeTXT) to simulate content that will be added to Actify! in future iterations. The cessation content integrated into future versions of the app could result in differential use and impact. Actify! also included very minimal coverage of two elements of BAT-D that were deemed by the team to be potentially problematic in the mHealth app format—hour-by-hour activity monitoring and social contracts. This decision was based on our early Actify! user research as well as our broader experience with health app development, which has suggested that programs that are too burdensome or inadequately sensitive to the social stigma still attached to mental health conditions (ie, lengthy, time-intensive self-monitoring prior to initiating activity planning; use of social contracts to engage the user’s social network) increase the risk of attrition. However, in future iterations of the app, we will continue to explore methods to improve self-awareness and social support while minimizing risk of attrition. Finally, our sample, on average, was of high socioeconomic status, had a low level of nicotine dependence, and only included smokers with mild to moderate depression; thus, results may not generalize to smokers who are more severely depressed, more nicotine dependent, and of lower socioeconomic status.

### Conclusion

While primarily designed to inform further refinements to Actify!, our findings from the user experience interviews contain important lessons learned that may be useful to others who are developing digital interventions based on BAT-D. For example, prior experience with in-person BAT-D interventions has demonstrated that the distinction between values and goals or activities is difficult for people to understand [10]. Based on our user experiences, this remains at least as challenging, if not more so, in self-guided mHealth interventions, and creative solutions are needed. This pilot trial also highlights the utility of implementing a user-centered design framework to develop mHealth interventions. Insights from posttreatment interviews indicate that the acceptability, and likely the outcomes, of Actify! were limited by usability issues not identified in our initial lab-based user testing phase. Future research could focus on allowing participants to use the app in their natural environment (eg, using diary study methodology to assess usability) to identify critical usability issues with activity planning and scheduling—core elements of the BAT-D treatment model—prior to a pilot trial. Nevertheless, the interview data gathered from pilot trial participants provide clear direction for the next iteration of the app. Key planned improvements include integrating therapeutic rationale and instructions for use into the main features of the app rather than in introductory onboarding screens, restructuring values identification to clarify the distinction between values and activities, and simplifying activity scheduling and tracking.

## Appendix

Multimedia Appendix 1 Primary and secondary user personas.

## Abbreviations

BADS: Behavioral Activation for Depression Scale
BAT-CS: Behavioral Activation Treatment for Cardiac Smokers
BAT-D: Behavioral Activation Treatment for Depression
BATS: Behavioral Activation Treatment for Smoking
CO: carbon monoxide
Fred Hutch: Fred Hutchinson Cancer Research Center
FTND: Fagerström Test for Nicotine Dependence
mHealth: mobile health
NCI: National Cancer Institute
PHQ-9: 9-item Patient Health Questionnaire
PPA: point prevalence abstinence
RCT: randomized controlled trial
TLFB: timeline follow-back
