# Supplementary figures and images for: A Behavioral Activation Mobile Health App for Smokers With Depression: Development and Pilot Evaluation in a Single-Arm Trial
Source: JMIR Form Res. 2019 Nov 27;3(4):e13728. doi: 10.2196/13728 (PMC6913543; doi:10.2196/13728)

Multimedia Appendix 1. Primary and secondary user personas.


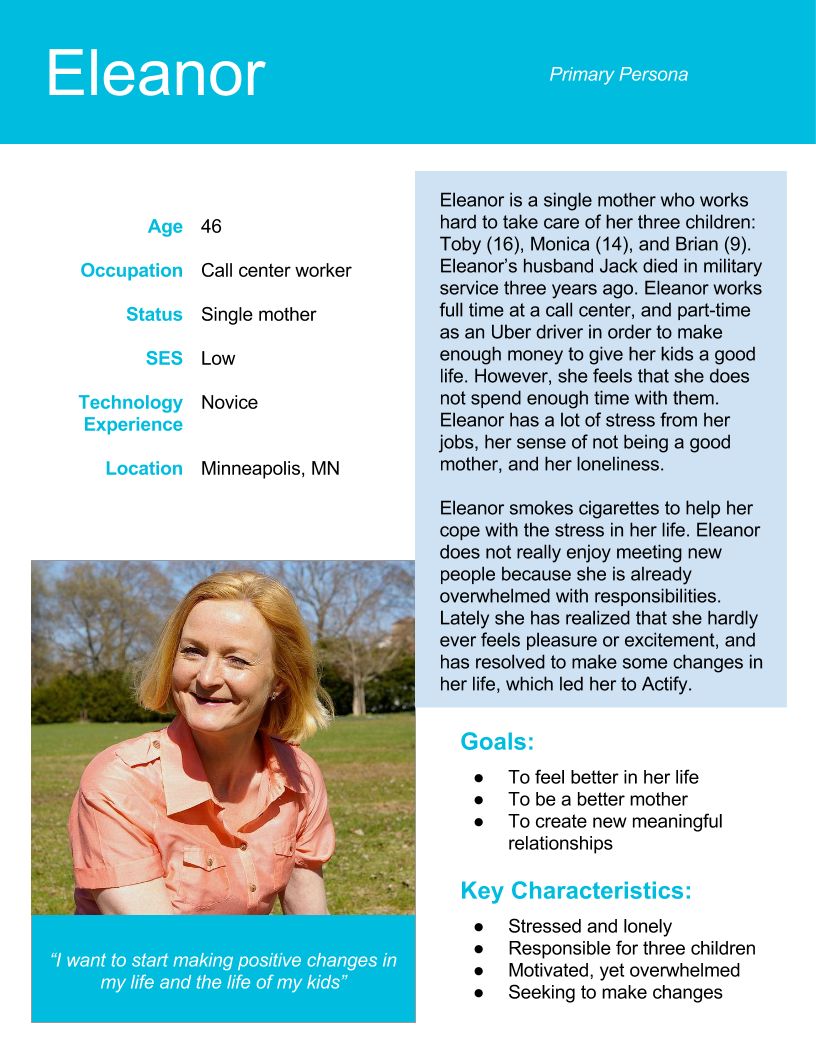


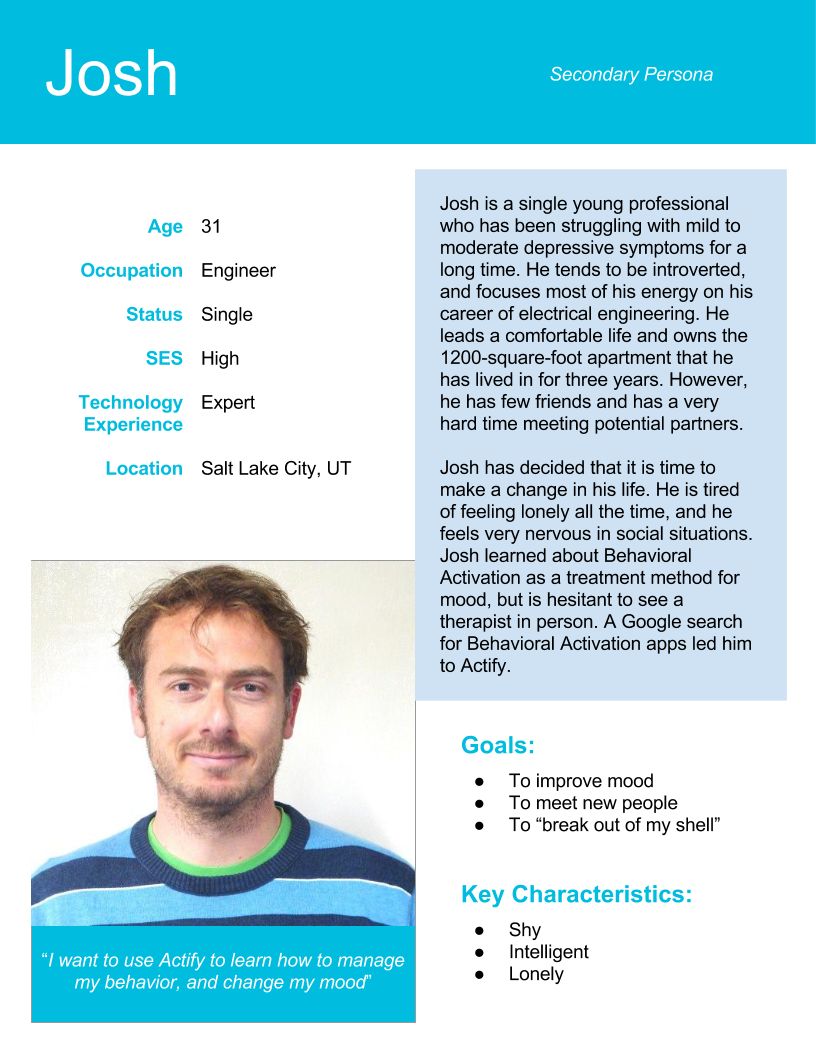

Supplement: Multimedia Appendix 1 [file formative_v3i4e13728_app1.docx]
